# On Diabetes

**Published:** 1822-06

**Authors:** 


### Art. V.—*On Diabetes.* By Dr. Trotter.

SINCE I had occasion to address you on the subject of *Diabetes Mellitus*, and the effect of *pure magnesia* in the cure of it,* three confirmed cases have come under my observation. The first, in a young man, G. L., æt. 28, who had possessed a fine person, being six feet two inches, and had enjoyed excellent health. The disease began in 1818; and, when he found himself too weak for his employment in a colliery as a driver, he was discharged. At the time he applied to me he was extremely emaciated, and with much difficulty walked to my house. From his appetite being so keen, he scarcely suspected himself labouring under disease; and his father and brother, with whom he lived, complained that he devoured all the food intended for them. His drink, in the day, now amounted to sixteen quarts; and the urine made much about that quantity. The bowels were slow, the skin dry, and the urine sweet as honey.

This poor fellow had only eighteen-pence a-week for his parish allowance, and I endeavoured, by representing his case, to get it increased; but the overseer would only consent to add a single shilling; so that the medicine which I wished him to pursue could not be tried. I then made his case known to one

* Vol. xxxix. p. 366.

of the coal-owners, that he might be received into the Infirmary; but he died before this could be accomplished.

2d.—The next case had a more fortunate issue. J. H. aged 50, a married woman, applied to me in November 1821. She was tormented with thirst; drank eight quarts of water, and made eight quarts of urine, in the day, of a peculiar smell, which grew frothy in a short time, and was quite like sugar to the taste. She was losing flesh, though she had a great appetite, and found herself growing weak. These complaints began about six months ago. She was ordered to take ℥iss of magnesia in twenty-four hours, which quickly opened her bowels; and the thirst ceased on the third evening; the water, at the same time, changed its taste and appearance, and the appetite became natural. The medicine was continued a week or ten days, and her health seemed quite restored; though her relatives had, at first, looked upon her case as deplorable.

3d.—The third case was that of a young woman of twenty-two, a servant. The disease commenced two years ago. She came to me in January last, and was considerably reduced. Having no relations, she was compelled to remain at service; but had now a humane mistress, who was kind to her, and, by a little charity from her physician, she was enabled to buy her medicine. The drink was about eight quarts in twenty-four hours; the urine rather more, sweet as honey, and of a frothy quality. The appetite voracious, and the bowels slow; the skin dry, and the thirst constant, night and day. The menses disappeared seven months ago. The magnesia, to the amount of two drachms in the day, was immediately begun; and on the second day the thirst declined, and next day the urine fell to three quarts, and nearly natural in taste and smell. The appetite also was less keen, and the bowels profusely loose. In a week or two she found her strength greatly improved, and can now go through her work without feeling fatigued. There is, as yet, no appearance of the menses: indeed, a complaint of such a nature, continuing for so long a time, could not fail to make serious inroads upon the constitution; and, when it arrives at a certain point, there can be no recovery. The quantity of urine is now two pints, and sufficiently saline; but, though diabetes may not return, there is still some doubt of perfect health.

I thought it superfluous to make any experiments on the blood and urine in any of these cases, as their appearance and nature were sufficiently ascertained before.

The diet, in all these persons, was the usual fare of labouring people; and the effect of the medicine seemed not to vary by any accidental change. That a diet entirely of animal matter should afford the saccharine quality to the urine, as in the first case transmitted to you before, would seem to contradict the experience of Dr. Rollo on this subject. I think, however, that eminent physician viewed the pathology of diabetes too much as a chemical doctrine. The omnipotence of a living principle in the system, is not to be denied; and, whatever processes resembling chemical combinations may be produced in the body, they can only be vicarious of this administering power. The purgative effect of magnesia, as exhibited in these patients, plainly shows a predominant acidity in the stomach and bowels: this is, probably, the *saccholactic* acid, and evolved in the process of deranged digestion. Many of the attendant symptoms can only be considered as sympathetic with this extraordinary condition of the digestive powers.

These five cases of diabetes are the proportion that I have met with in *ten thousand seven hundred persons* of the labouring class, to whom I have given medical advice since 1802; and many of them have come from very remote parts of this and the neighbouring counties. I have known about twenty instances of this disease in all; but, from all the circumstances of sex, age, temperament, season, situation, employment, habits of life, &c. I cannot form any peculiar character of predisposition that gives birth to its singular train of symptoms.

In my former communication, it was thought necessary to warn physicians against the adulterations of magnesia calcinata. What was employed so successfully by myself is the preparation of a London chemist. The purity is to be judged by the directions given by Henry to his own preparation, and I would take the article of that scientific chemist as a standard: it is only to be lamented that his price puts it beyond the reach of the poor patient. But, as pure magnesia is of such great value in the treatment of all diseases of what I designate by the name of *nervous temperament*, forming by far the largest class of modern ailments, I would recommend to the Royal College to petition Parliament for a full remuneration to Dr. Henry for his method of preparation, that the value of the medicine may be more generally known. I conceive the president and fellows of the Royal College of Physicians to be the proper organ for such an application.

The simplicity of the practice here announced in the treatment of diabetes, will be its recommendation to the ingenuous and disinterested part of the profession; and, to the confidence of their approbation, I leave the future trials of the medicine.

*Newcastle; May* 4, 1822.

